# Methodological development of the interactive INTERLINKS Framework for Long-term Care

**DOI:** 10.5334/ijic.1173

**Published:** 2014-06-23

**Authors:** Jenny Billings, Kai Leichsenring

**Affiliations:** Applied Health Research, Centre for Health Service Studies, University of Kent, Canterbury, Kent, UK; European Centre for Social Welfare Policy and Research, Vienna, Austria

**Keywords:** long-term care, older people, integrated care, systems theory, evidence

## Abstract

There is increasing international research into health and social care services for older people in need of long-term care (LTC), but problems remain with respect to acquiring robust comparative information to enable judgements to be made regarding the most beneficial and cost-effective approaches. The project ‘INTERLINKS’ (‘Health systems and LTC for older people in Europe’) funded by the EU 7th Framework programme was developed to address the challenges associated with the accumulation and comparison of evidence in LTC across Europe. It developed a concept and method to describe and analyse LTC and its links with the health and social care system through the accumulation of policy and practice examples on an interactive web-based framework for LTC. This paper provides a critical overview of the theoretical and methodological approaches used to develop and implement the INTERLINKS Framework for LTC, with the aim of providing some guidance to researchers in this area. INTERLINKS has made a significant contribution to knowledge but robust evidence and comparability across European countries remain problematic due to the current and growing complexity and diversity of integrated LTC implementation.

## Introduction

There is increasing international research into health and social care services for older people in need of long-term care (LTC), but challenges remain with respect to acquiring robust comparative information to enable judgements to be made regarding the most beneficial and cost-effective approaches [1–3]. It would seem that theories and methods to improve coordination have been proposed and implemented but remained patchy [2, 4–6] restricted in scope to disease management or pilot projects that are not always able to show clear evidence for improvements [7].

While this suggests that models for the identification of good practice across countries are still evolving [8], the complexity of undertaking research in this field cannot be overestimated. Authors such as Stein and Rieder [9] lament that, at the very least, there should be a common terminology by now upon which to build consistent methods and produce reliable comparative measures, but this fundamental aspect is an ongoing, largely unresolved issue, and this is only one of many challenges [7]. Researchers are grappling with differing health reforms and policy imperatives that may require a change of direction or emphasis during the lifetime of projects, restrictions to resource allocation that affect implementation of initiatives, and of course the increasing complexity of the health conditions suffered by a multi-morbid ageing population. All this has the potential to impact on the validity of the research process, and lead to the problems with comparability. Therefore, approaches to research in this field must be creative and innovative, and use a multiplicity of approaches.

The project ‘INTERLINKS’ (‘Health systems and LTC for older people in Europe’) was developed to address some of these methodological challenges. INTERLINKS was an EU-funded project (FP7 – Grant agreement no. 223037) conducted between 2008 and 2012 and was designed to expand on the INTERfaces and LINKS between prevention and rehabilitation, quality of care, informal care and governance, and to blend these single elements in a general framework for describing, comparing and analysing emerging LTC systems. The consortium represented 13 EU member states (Austria, Denmark, Finland, Germany, Greece, Italy, the Netherlands, the Slovak Republic, Slovenia, Spain, Sweden and the UK with a focus on England) as well as Switzerland covering different welfare regimes and geographical domains to allow for the regional differences to be addressed.

The main objectives of this project were to develop a concept and methodology to describe and analyse LTC and its links with the health and social care system, and to accumulate examples that illustrate key issues of policy and practice when developing integrated LTC systems focusing on informal care, prevention and rehabilitation, quality assurance as well as governance and financing.

The outcome of INTERLINKS was a web-based Framework for LTC constructed around relevant themes, sub-themes and 135 key issues that are illustrated by almost a hundred examples of validated practice in LTC for older people. This knowledge base intends to allow those working in the field to assess, compare and improve their own practice (see http://interlinks.euro.centre.org/).

While results and findings including a range of practice examples are published elsewhere [3] the purpose of this paper is to provide a critical overview of the theoretical and methodological approaches used to develop and implement the INTERLINKS Framework for LTC, with the aim of providing some methodological guidance to researchers in this area concerning design and application, cross-national collaboration and how to ensure robustness through validation and dissemination issues.

Given the difficulties in establishing sound evidence in LTC, much of the development reflected an amalgamation of inductive, experiential and tacit knowledge of LTC expertise, alongside existing theory and evidence from a variety of sources. This paper will provide an account and rationale for the approach, reviewing the extent to which it is providing a robust resource for policy and practice. It will commence with a description of how the methodological foundations of the INTERLINKS Framework were built and follow with an account of how the practice examples were compiled and validated, including how evidence was defined. The discussion will focus on a critical review of the approach and conclude with some recommendations for research practice.

## Building the foundations of the INTERLINKS Framework for LTC

The process of envisaging how a methodologically sound, comprehensive and universally understood framework could be developed to gather practice examples that could describe and analyse the complexities of LTC across Europe, commenced with the elaboration of two ‘vignettes’ describing and visualising different pathways of people in need of care in the participating countries. These vignettes were relevant in revealing the diversity and complexity of the different systems of integrated care across the EU. A series of discussion papers underpinned by policy, theory and research followed, the purpose of which was to disentangle thought processes in this complex and diverse area and gain consensus on fundamental principles in order to start the building blocks of framework conceptualisation. There was a consideration of systems theory [8], a conceptual analysis of boundary work [10] and elaboration on the place of person-centredness [11].

Following internal discussions, it was decided that systems thinking [12–14] was the most appropriate approach for developing a framework to promote the understanding of emerging LTC systems. Systems thinking is particularly useful because it helps identify the mutual interdependencies of stakeholders involved and of given contextual conditions as well as specific patterns of structures and processes in the planning, organising and monitoring of services and facilities. Finally, systems thinking lends itself very well to elaborate on complexity and related reduction of complexity [15].

Further to this, in the past decade a number of quality management models and frameworks have emerged [16] and these were considered and critically reviewed. They included the ‘Chronic Care Model’ (CCM) [17], and subsequent versions the ‘Innovative Care for Chronic Conditions Framework’ [18] and the ‘Expanded Care Model’ [19]; the ‘Guided Care’ model [20, 21], the ‘Evaluation Framework for disease management’ [22] and the ‘European Foundation for Quality Management Model’ (EFQM) [23] among others. On reviewing these models, only the EFQM quality management model and the CCM are frequently and internationally used, have assumed or proven relationships between the models components, and seem to bring about health care improvements. However, these models do not consider the complexity of LTC and the systems approach sufficiently well, lack specific details of tackling interfaces and links between organisations and thus do not have integrated care as a predominant perspective. For example, the EFQM quality management model primarily concentrates on the dynamics within organisations and not on interorganisational care pathways. Furthermore, while the CCM is valuable, it is aimed at chronic patient groups rather than broader LTC service provision, as are others [22].

More recently, Minkman et al. [5] have created and implemented a ‘Development Model for Integrated Care’, consisting of 89 elements of integrated care grouped in nine clusters (patient-centredness, delivery system, performance management, quality of care, result-focused learning, interprofessional teamwork, roles and tasks, commitment and transparent entrepreneurship). Despite its application on the specific conditions of stroke, acute myocardial infarction and dementia, aspects of Minkman et al.'s work were drawn upon due to its firm focus on integration and broader appeal to our approach.

These first developments built on a pragmatic and visual view as to how INTERLINKS knowledge should be grouped and presented, setting out from the start how the results of the project could be configured as a web-based tool. Some underlying principles were agreed deriving from the theoretical debates. It was suggested that the framework should be logical, evidence-based and reflect the individual, professional, organisational and systems levels, and that it should focus fundamentally on and be underpinned by ideal pathways of the individual client. Further to this and connected to the underlying ethos of previously reviewed models, there was agreement that our framework should recognise that care provision and support should be continuous and cohesive, empower clients and strengthen their self-direction, which is key to maintaining quality of life [24–26].

It should also reflect a human functioning perspective that is applicable to older frail and dependent people; for example, that frail older people using LTC have multiple needs which must be holistically addressed in their own context.

**Figure 1.**
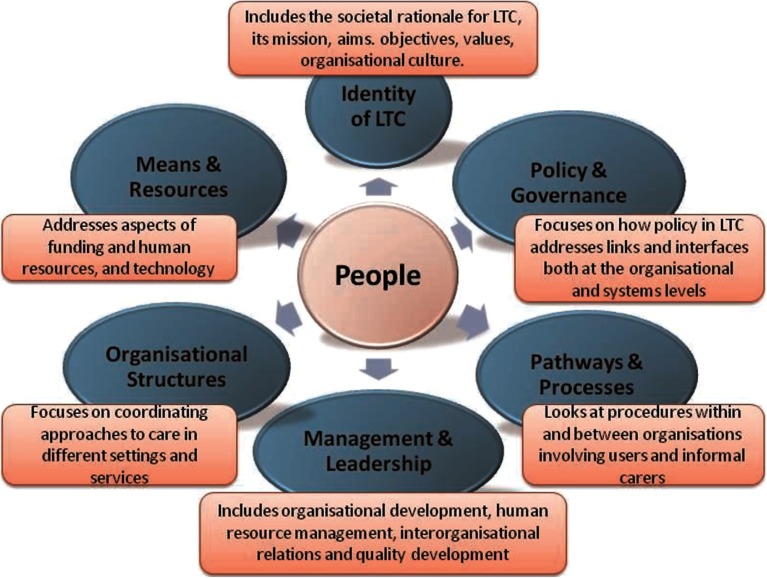
The themes for the INTERLINKS Framework for LTC

This structure went on to be developed in more detail during an iterative process, adapting and evolving according to partner discussions, a validation process, and as practice examples were being gathered. A final framework was developed which met the aim of illustrating what needed to be in place to address the links and interfaces between social and health care systems, and formal and informal care, and was finally structured around six main, but non-hierarchical themes that correspond to the most important features of a virtual LTC system with its interlinkages described in Figure 1.

**Figure 2.**
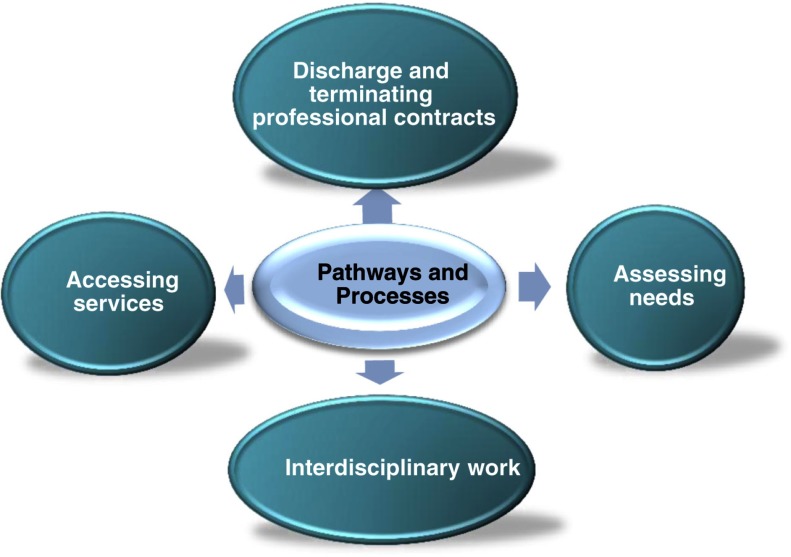
Subthemes within ‘Pathways and Processes’

There are three layers to provide the complex details within the framework, namely themes, subthemes and key issues. Each theme was further defined by a number of subthemes that, again, were further specified by relevant key issues. These numerous specifications were again developed through comprehensive use of findings across Europe accumulated through previous INTERLINKS research and through protracted internal discussions and external validation. Figure 2 below gives an illustration of subthemes within the theme of ‘Pathways and Processes’.

**Figure 3.**
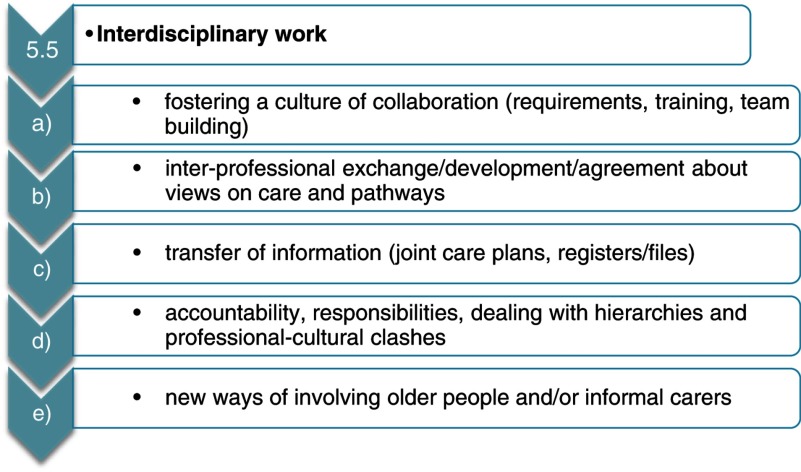
Key issues within subtheme ‘Interdisciplinary work’

In turn, Figure 3 goes to the next layer of detail and gives an example of the key issues partners needed to search for when compiling practice examples for ‘Interdisciplinary work’.

Systems theory was valuable in enabling the harmonisation of multi-layered, detailed aspects of LTC provision at the system, organisation, care delivery and older person/informal carer levels to come together for the framework. Thus, the framework provided the necessary guidance for partners to identify and collect selected practice examples to illustrate individual key issues with a focus on the links and interfaces or respective gaps.

## Compilation and validation of practice examples

### Defining evidence

An important part of developing a useful and valid database of practice examples would depend upon the nature of the evidence accumulated. It was felt important from an early stage therefore to set out a definitive position on what constituted evidence within INTERLINKS and in general in the area of LTC. This debate had to take into account that, as LTC systems are only emerging across Europe, so does related research and knowledge production. The following provides a succinct rationale for adopting a *pluralist approach* to evidence accumulation by recognising ‘other forms of evidence that require tolerance of epistemological diversity in order for the full range of research evidence to be utilised’ [27].

Evidence-based practice has been recognised and embedded into health and social care policy and practice across Europe to varying degrees only recently, with the understanding that with this comes a set of assumptions about what constitutes evidence [28, 29]. It is well recognised that there exists a hierarchy, from ‘gold standard’ controlled trials through to evidence derived from more experiential sources [30].

For a while now, the need for a broader view of what constitutes evidence is being vocalised by health and social care academics, as traditional views of evidence do not account for the complexities of policy generation and practice needs [31]. Moriarty et al. [32], for example, argue that the reality is that professionals and policy-makers have always needed to draw upon diverse sources of information when making decisions about complex health and social care issues. This includes not only evidence from research, but also other more qualitative evidence stemming from value judgements such as public preferences for a particular intervention or approach to care [33].

Projecting this into the arena of LTC for older people, it is clear that a pluralist framework of evidence fits keenly with the evidence requirements of this area, particularly in relation to the types of knowledge needed for good practice. Cheetham et al. [34] and Petch et al. [35], for example, stress the importance of a multi-perspective approach to understanding what constitutes meaningful evidence and outcomes in LTC. This includes a move away from the narrow range of indicators towards organisational, professional and user inputs, making visible important factors that contribute towards a more appropriate evidence base. This ranges from policy to case studies of good practice. Evidence from PROCARE [36] and EUROFAMCARE [37] has also done much to contribute towards this.

All the while that uniquely quantitative designs are relied upon, the evidence for LTC will remain elusive and incomplete. Therefore, a wider spectrum of evidence including qualitative sources needs to be embraced in order to be able to develop methods that can fully describe and analyse the multifaceted and complex system that is LTC, hence the approach adopted in this study.

### Data collection and validation

A relevant aspect to consistently collect and analyse practice examples was to create a structured form to illustrate the 135 identified key issues of LTC systems. In terms of structural details, the form needed to be reliable and understandable, and permit the consistent collection of detailed practice examples from partner countries using a plurality of evidence sources, in order to support the development of a database of comparative European policy and practice.

**Figure 4.**
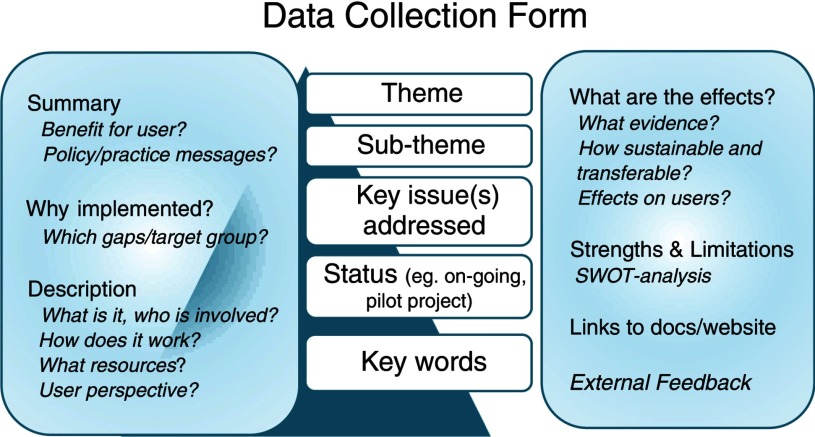
Data collection form

Figure 4 illustrates the eventual structure for the form. Important features included detailed instructions concerning what should be included to promote consistency and reliability of information. Emphasis was given to ensure that practice examples revealed the user and carer perspective throughout, and also addressed the links and interfaces both present and missing. A consideration of how the information would be best presented to attract and hold the attention of a web audience of practitioners and policy-makers, also resulted in making prominent certain information, such as the inclusion of simplified headings for the sections, a summary, prominent messages to practitioners, client and user benefits, weblinks to documents, and keywords and credits for the peer-reviewing process, as more experts external to the project team took part in this.

Parallel to this, a set of criteria were composed to guide the selection of practice examples (see Table 1).

Validation of the methodological processes and practice examples took place at three levels. First, the internal validation process was organised between INTERLINKS partners through a peer-review process by giving clear, unambiguous and detailed instructions with review questionnaires. Second, external validation took place with National Expert Panels (NEPs) – a sounding board (20 European experts from a wide range of organisations such as the Social Platform and European Federation of Older People, Alzheimer Europe, EUCOMED, EUROCARERS, European Social Network [ESN], Home Care Europe, AGE – European Older People's Platform, and the European Association for Directors of Residential Care Homes for the Elderly [EDE]) – and external communities interested in LTC to validate the consistency of elements and key issues of the INTERLINKS Framework and the illustrating practice examples.

All comments derived from consultations provided an important steer regarding the subsequent and final development of the main INTERLINKS outputs. For example, work was undertaken to increase and improve the visibility of older people and their carers through elaboration on the template and within the key issues.

Third, web-based engagement was planned. A primary intention of the website has been to attract other interested communities and gain their feedback about the examples, in order to promote a broader inclusive validation and generate consensus about the potential of the examples to apply in different areas. Considerable work has been done to ensure ease of access to examples and other information. In addition, external users of the website have been encouraged to add their own practice examples. However, one year on it must be noted that despite high numbers of visitors, the type of European interaction that was planned for has not taken place, an aspect discussed in the next section.

## Discussion

This section will critically discuss the processes described above, looking at the extent to which this methodological approach was sufficiently rigorous, and provided a robust and comparable resource for LTC.

Overall, the project succeeded in creating a detailed product for LTC systems’ assessment and improvement focusing on the gaps and interfaces between health and social care, and informal and formal care. Out of necessity, the framework became highly structured in order to achieve consistency of data collection. Much time was allocated to reaching consensus about the finer details of selection criteria, guidelines, lists of subthemes and key issues, and ensuring that project linguistics was jointly understood. Added to this, the development reflected an amalgamation of inductive, experiential and tacit knowledge of LTC expertise within and outside of the consortium, alongside existing theory and evidence from a variety of sources. The importance of tacit knowledge to build health and social care theory has been strongly debated for a number of years [31, 38]; therefore, inclusion was felt to be justified. So the project embraced a multi-method and consensual perspective not only with the collection of evidence from practice examples, but also in its construction. As a result, the wide range of examples was able to reveal beneficial innovations in a number of areas. This included technological applications to improve integrated care and support independence [39], the value of volunteering in LTC [40] and efforts in quality management that impact on quality of care and patient safety [41].

In addition, the framework was able to indicate where there are paucities of information. It became evident, for example, that there is a continued need to enhance prevention and rehabilitation opportunities *within* LTC across Europe [42]. Interestingly, examples of management and leadership in LTC are not plentiful, which might be an indicator for a lack of innovators and bridge-builders in this area. Added to this, the database of examples reflected a focus on single diseases such as stroke and Alzheimer's disease. This singular focus has implications for the move towards care provision to deal with people with co-morbidities, such as through ‘comprehensive care models’ [6], case management [43] and navigation roles [44]. The increase in research needed to address co-morbidity once more raises the potential for mixed-method strategies to disentangle the complexities of these approaches to reveal success factors [7].

The INTERLINKS framework for LTC does continue to demonstrate how the intricacies of integrated LTC delivery trigger a variety of different and relevant approaches, alongside innovative practice in specific areas. The pluralist perspective encouraged this diversity to a large extent; but this outcome does raise questions regarding meaningful comparability in a situation where ample differences between countries’ state of LTC development remain evident. Examples revealed that in some cases, there were difficulties transferring results between regions in the same country, despite evidence of effectiveness. In addition, there was some northern and southern European imbalances between numbers of practice examples collated, with less from southern Europe. Garcés et al. [45] note that this may not only be due to data availability, but also point out that while care services have similar aims across Europe, implementation in the North is greater than the South. However, the detail within the examples as a whole has permitted LTC principles of project set-up, implementation, costs and, for some, evaluation to be made evident. So INTERLINKS has certainly created a forward movement in how practice and policy in the six thematic areas of the framework can be compared across Europe.

On the whole, the validation process built within the project served to engender greater credibility and face validity within the research process. It has to be noted that although the internal consortium consisted of a large number of LTC experts from across Europe and provided much expertise and knowledge, it was vital to include an external validation component. Drawing on the knowledge of experts disconnected to the project provided an important objective, critical and emotionally detached edge that improved the credibility of the outcome. Such an inclusion is one which is increasingly used as a way of ensuring processes and outputs are convincing. Korpela et al. [46], for example, described a successful collaboration process for creating a valid roadmap for implementing a strategy for integrated health and social care in Finland. Also, a PROGRESS project coordinated by the European Centre [47] (see also [48]) developed a validation process through a mix of Delphi consultations, validation workshops with key stakeholders and a consensus group of research network stakeholders, to create a common view on relevant performance indicators in care homes. However, there were challenges to this process within INTERLINKS that needed to be addressed methodologically. With individual experts having their own views on the way forward, it became difficult to capture and collectivise the comments into actual changes at times.

A key feature of the project was to accumulate examples of good practice, verified as far as possible by robust evaluation, in order to contribute towards the lack of evidence hitherto. An additional facet of INTERLINKS was therefore to undertake an examination of those examples, assessing them for scientific merit. A total of 25 examples had a practice component amenable to evaluation, and had used mixed methods or single method evaluation of scientific merit [7]. These examples also produced impacts that could be generalised or used for local improvement. It was of interest that 30 examples had incomplete, minimal or no evaluation, but nevertheless their approaches and tools had been rolled out or were ongoing. These examples included the large scale implementation of policy imperatives, such as hospital discharge lounges in the UK [49], and the perceived urgency for an intervention, such as a needs assessment initiative in Slovakia [50]. Although the INTERLINKS examples may not be representative of all European countries, this investigation supports previous commentary that suggests that the evidence on LTC continues to be elusive [51–53].

Although the website has been visited by more than 5000 users during the first two years, a less successful feature concerned the ambitious desire for an interactive, web-based engagement from interested communities. This may be partly due to language issues, but we must also question the accessibility and complexity of the information and its subsequent transferability to different practice contexts. When considering how practitioners and policy-makers effectively seek and access information relevant to their needs, it cannot automatically be assumed that the internet satisfies all requirements. While there seems to be a move away from the tangible to the virtual in terms of information provision, clear solutions for research dissemination to appropriate target groups are elusive and tend to remain largely ‘guesswork’.

With this in mind and against the backdrop of increasing health and social service restraints across Europe, it is however important to question the extent to which the INTERLINKS framework will support fundamental and sustainable changes to integrated LTC provision. The interactive database has created some interest among the research community, but has yet to make a recognisable impact. As intimated, implementing and evaluating integrated care continues to be challenging; there were examples of projects from INTERLINKS that were not sustained outside of the project area, not mainstreamed, nor unable to demonstrate significant results.

INTERLINKS does make a contribution, through first revealing more unpublished projects throughout Europe, and making associated information more connected, accessible and visible; and second, enabling evidence to be generated and revealed within certain topic areas, such as the need for developing an own identity of LTC, multidisciplinary team approaches, quality assurance and quality improvements, the integration of informal carers, and innovative approaches in integrating palliative care [3]. The comprehensive framework for LTC will also be an important instrument to be used in relevant education and further training to promote the development of consistent LTC systems.

Indeed, and contrary to current frameworks that were developed in the context of health care delivery previously, the ambitions of INTERLINKS capitalised on the arguments put forward already by Ikegami and Campbell ([15] p. 730) who emphasised that ‘establishing a new LTC system (…) has appeared to be the best or only solution to major social policy issues’. A step forward has certainly been made by providing a comprehensive framework that helps identify and analyse the complex issues that have to be considered when constructing such a system.

When Somme and de Stampa [54] analysed 10 years of integrated LTC care for older people in France, they concluded that there is some movement from a linkage-based model to an integrated care system, but this is still ‘work in progress’. This is clearly compatible with INTERLINKS findings, but it is also evident that interested research and policy groups are far from giving up. Further strides are being made, particularly through the work of the King's Fund [55, 56], alongside others that seek to undertake transformation change in LTC [5, 57], and endeavours at the EU level such as the EU's Innovation Partnership for Active and Healthy Ageing, within which ‘integrated care’ is one of the key topics for action.

## Conclusions and recommendations

The aim of this paper has been to provide a critical overview of the theoretical and methodological approaches used to develop and implement the INTERLINKS Framework for LTC, with the aim of providing some methodological guidance to researchers in this area.

Four main points are offered here. First, INTERLINKS has demonstrated that there is a need to examine the value of different designs and to determine the extent to which they can best capture the effects of innovative LTC developments. This should include, for example, a move towards a broader multidisciplinary orientation and consider pluralistic and technological approaches that incorporate a more lateral spectrum of data collection techniques and data sources. Second, in the face of linguistic differences and understanding, it is vital to develop a sound set of detailed tools, selection criteria and guidelines underpinned by a plurality of knowledge and consensus between partners, to ensure the reliability and validity of data. It is important not to underestimate the time it takes to undertake these methods processes. Third, there is considerable value in building a realistic and robust validation process into the methods design, to ensure relevance and applicability within countries and importantly to countries lying outside of the project consortium. To this end, a very practical recommendation is to ensure agreement is reached regarding expectations of involvement so as not to overburden experts, and also to create guidelines for how their feedback is handled and included in the project.

Lastly, the inclusion of a web design for the eventual product such as the INTERLINKS Framework for LTC is recommended as it creates an output which is immediately accessible during the project lifetime and counters criticisms levelled at research in that it lags behind practice requirements. This must be considered in the early project phases so that project thinking is always aligned to a practical web-based outcome. While our database did not create the desired interaction, it has certainly courted a reasonable number of visitors.

INTERLINKS has certainly contributed to the ongoing epistemological debate on integrated LTC, adding to the current mainstream discussions on disease management and integration processes within the health care system, including related criticism when it comes to LTC for older people with multi-morbidities [58]. The complexity of LTC identified by the multiple key issues may mean that the level of reflection, analysis and cross-country translation required for comparative studies might be too challenging. The final publication of INTERLINKS [3] has therefore made in-roads into how individual solutions can be monitored and compared, and how organisations and policy-makers have successfully addressed a range of problems that will remain relevant in the foreseeable future.

## Figures and Tables

**Table 1.**
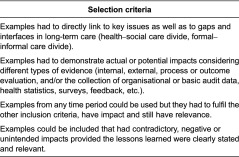
Selection criteria

## References

[1] Johri M, Beland P, Bergman H (2003). International experiments in integrated care for the elderly: a synthesis of the evidence. International Journal of Geriatric Psychology.

[2] Strandberg-Larsen M, Krasnik A (2009). Measurement of integrated healthcare delivery: a systematic review of methods and future: research directions. International Journal of Integrated Care [serial online].

[3] Leichsenring K, Billings J, Nies H (2013). Long-term Care in Europe: improving policy and practice.

[4] Dickinson H, Glasby J, Glasby J, Dickinson H (2009). Introduction. International perspectives in health and social care.

[5] Minkman MMN, Vermeulen R, Ahaus K, Huijsman R (2011). The implementation of integrated care: the empirical validation of the development model for integrated care. BMC Health Services Research.

[6] De Bruin SR, Versnel N, Lemmens LC, Molema CCM, Schellevis FG, Nijpels G (2012). Comprehensive care programs for patients with multiple chronic conditions: a systematic literature review. Health Policy.

[7] Billings J, Leichsenring K, Billings J, Nies H (2013). Improving the evidence base. Long-term Care in Europe: improving policy and practice.

[8] Leichsenring K (2012). Perspectives: integrated care for older people in Europe – latest trends and perceptions. International Journal of Integrated Care.

[9] Stein KV, Rieder A (2009). Integrated care at the crossroads – defining the way forward. International Journal of Integrated Care [serial online].

[10] Gobet P, Emilsson T, Leichsenring K, Billings J, Nies H (2013). Integration as ‘boundary redefinition process’. Long-term Care in Europe – improving policy and practice.

[11] Billings J, Leichsenring K, Wagner L, Leichsenring K, Billings J, Nies H (2013). Addressing long-term care as a system. Long-term Care in Europe – improving policy and practice.

[12] Watzlawick P (1976). Wie wirklich ist die Wirklichkeit? [How real is reality?].

[13] Senge PM (1990). The fifth discipline. The art and practice of the learning organisation.

[14] Bateson G (2000). Steps to an ecology of mind.

[15] Ikegami N, Campbell JC (2002). Choices, policy logics and problems in the design of long-term care systems. Social Policy and Administration.

[16] Nolte E, McKee M, Nolte E, McKee M (2008). Integration and chronic care: a review. Caring for people with chronic conditions: a health system perspective. WHO/European Observatory on Health Systems and Policies.

[17] Wagner EH (1998). Chronic disease management: what will it take to improve care for chronic illness?. Effective Clinical Practice.

[18] Wagner EH, Austin BT, Davis C, Hindmarsh M, Schaefer J, Bonomi A (2001). Improving chronic illness care: translating evidence into action. Health Affairs.

[19] Barr V, Robinson S, Marin-Link B (2003). The expanded chronic care model: an integration of concepts and strategies from population health promotion and the chronic care model. Hospital Quaterly.

[20] Boult C, Reider L, Frey K, Leff B, Boyd C, Wolff J (2008). Early effects of “guided care” on the quality of health care for multimorbid older persons: a cluster-randomized controlled trial. Journal of Gerontology.

[21] Boyd C, Sadmi E, Jackson Conwell L, Grisworld M, Leff B, Brager R (2008). A pilot test of the effect of guided care on the quality of primary care experiences for multimorbid older adults. Journal of General Internal Medicine.

[22] Lemmens K (2009). Improving chronic care. Developing and testing disease-management interventions applied in COPD care. Dissertation.

[23] (2003). European Foundation for Quality Management: EFQM Excellence Model. Public and Voluntary Section Version.

[24] Kane RA (2001). Long-term care and good quality of life. Bringing them closer together. The Gerontologist.

[25] Schalock RL, Brown R, Brown RA, Cummins DF, Matikka L, Keith KD (2002). Conceptualization, measurement, and application of quality of life for persons with intellectual disabilities: report of an international panel of experts. Mental Retardation.

[26] Hollander MJ, Prince M (2007). Organising healthcare delivery systems for persons with ongoing care needs and their Families: a best practice framework. Healthcare Quarterly.

[27] Lambert H (2012). Plural forms of evidence in public health: tolerating epistemological and methodological diversity. Evidence & Policy.

[28] French P (2002). What is the evidence on evidence-based nursing? An epistemological concern. Journal of Advanced Nursing.

[29] Olsson TM (2007). Reconstructing evidence-based practice: an investigation of three conceptualisations of EBP. Evidence and Policy.

[30] Rycroft-Malone J, Seers K, Titchen A, Harvey G, Kitson A, McCormack B (2004). What counts as evidence in evidence-based practice?. Journal of Advanced Nursing.

[31] Glasby J, Beresford P (2006). Who knows best? Evidence-based practice and the service user contribution. Critical Social Policy.

[32] Moriarty J, Manthorpe J, Iliffe S, Rapaport J, Clough R, Bright L (2007). Promoting the use of diverse sources of evidence: evaluating progress in the provision of services for people with dementia and their carers. Evidence and Policy.

[33] Mays N, Pope C, Popay J (2005). Systematically reviewing qualitative and quantitative evidence to inform management and policy-making in the health field. Journal of Health Services Research and Policy.

[34] Cheetham J, Fuller R, McIvor G, Petch A (1992). Evaluating social work effectiveness.

[35] Petch A, Cook A, Miller E (2005). Focusing on outcomes: their role in partnership and practice. Journal of Integrated Care.

[36] Billings J, Leichsenring K (2005). Integrating health and social care services for older persons. Evidence from nine European countries.

[37] Mestheneos E, Triantafillou J (2005). Supporting family carers of older people in Europe: empirical evidence, policy trends and future perspectives. The Pan-European Background Report.

[38] Benner P (1984). From novice to expert: excellence and power in clinical nursing practice.

[39] Billings J, Carretero S, Kagialaris G, Mastroyiannakis T, Meriläinen-Porras S, Leichsenring K, Billings J, Nies H (2013). The role of information technology in LTC for older people. Long-term Care in Europe – improving policy and practice.

[40] Repková K, Stiehr K, Weigl B, Leichsenring K, Billings J, Nies H (2013). Volunteering in long-term care for older people: the potential for social innovation. Long-term Care in Europe – improving policy and practice.

[41] Leichsenring K, Nies H, van der Veen R, Leichsenring K, Billings J, Nies H (2013). The quest for quality in long-term care. Long-term Care in Europe – improving policy and practice.

[42] Kümpers S, Ruppe G, Wagner L, Dieterich A, Leichsenring K, Billings J, Nies H (2013). Prevention and rehabilitation in long-term care. Long-term care in Europe – improving policy and practice.

[43] Lupari M, Coates V, Adamson G, Crealey GE (2012). We're just not getting it right’ – how should we provide care to the older person with multi-morbid chronic conditions?. Journal of Clinical Nursing.

[44] Manderson B, Mcmurray J, Piraino E, Stolee P (2012). Navigation roles support chronically ill older adults through healthcare transitions: a systematic review of the literature. Health and Social Care in the Community.

[45] Garcés G, Ródenas F, Hammar T, Leichsenring K, Billings J, Nies H (2013). Converging methods to link social and health care systems and informal care – confronting Nordic and Mediterranean approaches. Long-term Care in Europe – improving policy and practice.

[46] Korpela J, Kaarna T, Elfvengren K, Tepponen M, Tuominen M (2012). Collaboration process for integrated social and health care strategy implementation. International Journal of Integrated Care.

[47] European Centre for Social Welfare Policy and Research (Coordinator) (2010). Measuring progress: indicators for care homes.

[48] Hoffmann F, Leichsenring K (2011). Quality management by result-oriented indicators: towards benchmarking in residential care for older people.

[49] Holdsworth L (2011). Hospital discharge lounges.

[50] Brichtova L (2011). A new national model of needs assessment implemented by local governments. http://interlinks.euro.centre.org/framework.

[51] Glasby J, Walshe K, Harvey G (2007). What counts as ‘evidence’ in ‘evidence-based practice’?. Evidence and Policy.

[52] Ramsay A, Fulop N (2008). The evidence base for integrated care.

[53] Petch A (2009). The evidence base for integrated care. Journal of Integrated Care.

[54] Somme D, de Stampa M (2011). Ten years of integrated care for the older in France. International Journal of Integrated Care.

[55] Curry N, Ham C (2010). Clinical and service integration: the route to improved outcomes.

[56] Appleby J, Harrison T, Hawkins L, Dixon A (2012). Payment by results. How can payment systems help to deliver better care?.

[57] De Manuel E New funding and commissioning model as a driving force for the transformation of the Basque health system provision model.

[58] Hughes LD, McMurdo MET, Guthrie B (2013). Guidelines for people not for diseases: the challenges of applying UK clinical guidelines to people with multimorbidity. Age Ageing.

